# Pulmonary Embolism: A Rare Presentation of Acute-on-Chronic Pancreatitis in Children

**DOI:** 10.7759/cureus.76768

**Published:** 2025-01-01

**Authors:** Nazia Iqbal, Maryam Mazhar, Sobia Noureen, Eman Shujaat, Tahir Malik, Iqtadar Seerat

**Affiliations:** 1 Department of Paediatrics Gastroenterology & Hepatology, Pakistan Kidney and Liver Institute, Lahore, PAK; 2 Department of Paediatric Gastroenterology & Hepatology, Pakistan Kidney and Liver Institute, Lahore, PAK; 3 Department of Radiology, Pakistan Kidney and Liver Institute, Lahore, PAK

**Keywords:** chronic pancreatitis, deep venous thrombosis, dyspnoea on exertion, multidisciplinary team, pulmonary embolism

## Abstract

Although splanchnic vein thrombosis may manifest as pancreatitis, it is rare to find venous thrombosis at other body sites. We present a rare case of acute-on-chronic pancreatitis in a patient who subsequently developed a deep venous thrombosis (DVT) of the right lower limb and pulmonary embolism (PE). A 14-year-old male presented with an acute history of upper abdominal pain, vomiting, and abdominal distension. Workup for pancreatitis was normal except for low hemoglobin and high serum pancreatic enzymes. The CT abdomen favored a diagnosis of acute-on-chronic pancreatitis with dilated pancreatic duct, multiple pseudo-pancreatic cysts, and saddle pulmonary embolus. He was treated with medical conservative therapy. For DVT and saddle PE, he was started on enoxaparin, which was later switched to rivaroxaban. A CT angiogram after three months of therapy with rivaroxaban showed the complete disappearance of the clot. Early detection of such life-threatening complications is important to achieve favorable outcomes in these patients.

## Introduction

Chronic pancreatitis (CP) is a chronic, slowly progressive, persistent inflammation of the pancreas, characterized by abdominal pain consistent with pancreatic origin or endocrine/exocrine pancreatic insufficiency and suggestive pancreatic imaging findings [1]. Although CP is rare in children and adolescents, it is associated with a significant disease burden and a negative impact on quality of life. This report highlights the importance of the involvement of a multidisciplinary team in the management of patients with acute-on-chronic pancreatitis to ensure optimal outcomes [2]. Pancreatitis complicated by pulmonary embolism (PE) is extremely uncommon, and the mortality rate is extremely high if not detected promptly. The thrombus causing PE mainly stems from deep vein thrombosis (DVT). It is currently believed to be mainly caused by systemic inflammation and blood hypercoagulability [3-5]. To decrease the risk of developing this very rare complication of thromboembolism, keen awareness and early recognition are important, and clinicians should promptly refer such patients to a tertiary center.

## Case presentation

A 14-year-old male presented to the outpatients clinic of Pakistan Kidney and Liver Institute in May 2023 with a history of recurrent severe epigastric abdominal pain along with frequent non-bilious vomiting, loose stools, significant weight loss, and abdominal distention for the past three months. He was referred to our outpatient department due to the persistence of his symptomatology.

**Figure 1:**
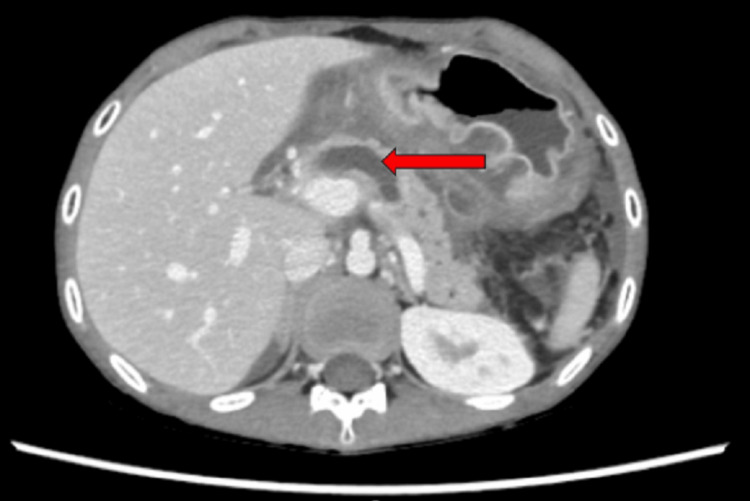
CT scan showing dilated pancreatic duct (arrow) CT: computed tomography

**Figure 2:**
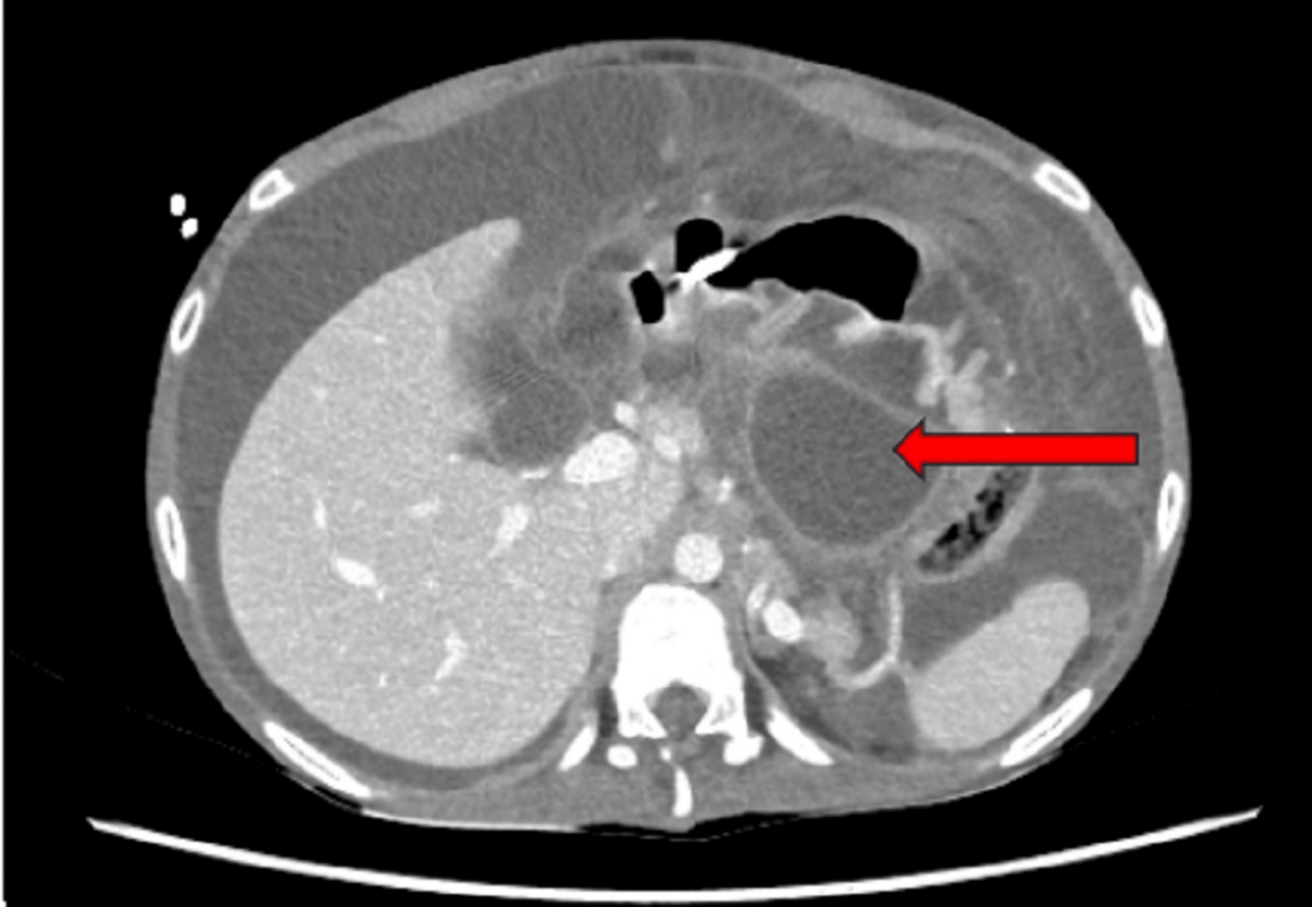
Abdominal CT scan showing pseudo-pancreatic cyst (arrow) CT: computed tomography

**Figure 3:**
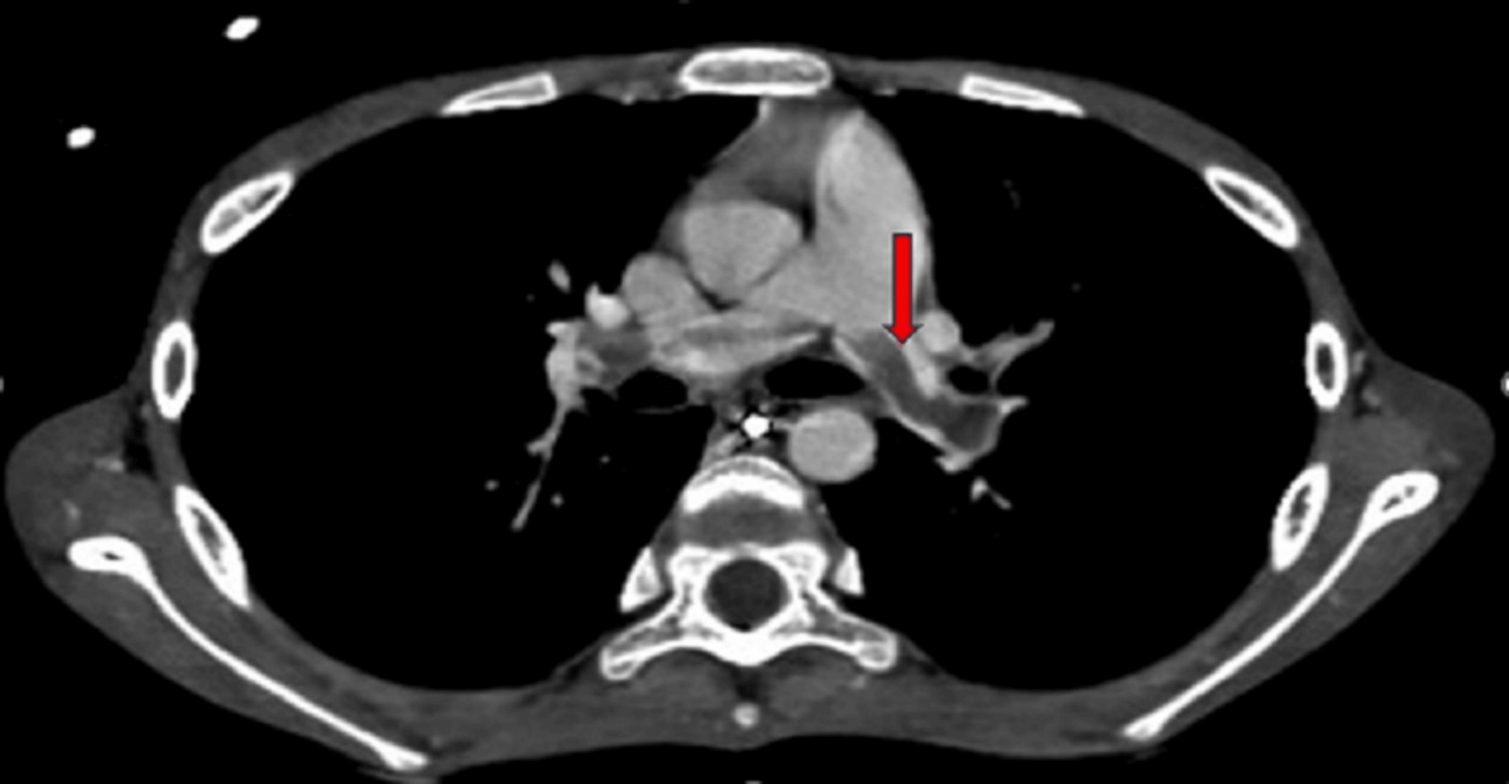
Abdominal CT scan showing saddle embolism in pulmonary artery and its branches (arrow) CT: computed tomography

On examination, he was a thin, emaciated boy whose weight was on the fifth centile and height was on the 90th centile; he had pallor, generalized reduced muscle bulk, and loss of subcutaneous fat. The abdomen was tender with gross ascites but no visceromegaly. The rest of the systemic examination was unremarkable. The baseline investigations, serum amylase, lipase, lipid and autoimmune profile, immunoglobulin levels, calcium, and viral markers were sent. The abdominal CT scan depicted acute-on-chronic pancreatitis with a dilated pancreatic duct (Figure 1) and multiple peripancreatic fluid-filled pseudo-pancreatic cysts (Figure 2), the largest measuring 7.5 x 8 cm with features of saddle PE (Figure 3).

**Figure 4:**
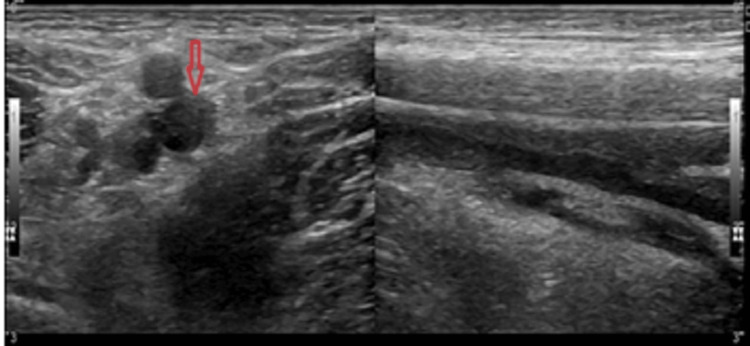
Doppler ultrasound showing thrombosed femoral vein (arrow)

Furthermore, a Doppler ultrasound of the extremities showed DVT in the right lower limb (Figure 4), and the D-dimer level was elevated. On exploring further, the patient reported often experiencing dyspnoea on exertion.

**Figure 5:**
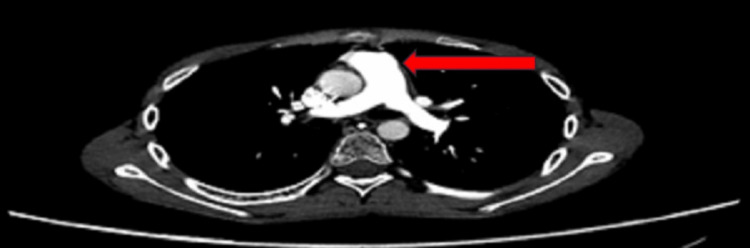
CT angiogram showing post-treatment disappearance of PE (arrow) CT: computed tomography; PE: pulmonary embolism

Based on this clinical presentation and investigations, a diagnosis of acute-on-chronic pancreatitis was entertained. The patient was started on treatment with a fat-free diet, antibiotics, octreotide for ascites, pancreatic enzymes, and analgesics. A nasojejunal tube (NJ) and ascitic drain were placed. Hydrolyzed milk bolus feeds via the NJ tube were given. Enoxaparin was started after hematological consultation based on DVT and saddle PE. His symptomatology, ascites, body weight, and laboratory investigations improved significantly after the treatment (Table 1), followed by the disappearance of the pancreatic pseudocyst, ascites, DVT, and PE (Figure 5) over three months. He continued to receive supportive care, including adequate caloric intake, after discharge and during follow-up, and his laboratory and radiological parameters were monitored.

**Table 1:** Improvement in weight and lab parameters after treatment

Investigations	Pre-treatment	Post-treatment	Normal value
Weight, kg	22	26	
Haemoglobin, g/dL	9	11.6	11.5-14
Platelets, x 10⁹/L	565	416	150-450
White blood cells, x 10⁹/L	7.7	8	4.6-11
Lipase, U/L	770	65	13-60
Amylase, U/L	1379	250	11-54
Albumin, g/dL	1.90	4.19	3.5-5
D-dimers, ng/ml	29,998	750	100-500
Sodium, meq/l	131	138	135-145
Calcium, mg/dL	8	9.75	8.4-10.2
C-reactive protein, mg/dL	3.09	0.50	<0.5

## Discussion

In this report, we describe a case of acute-on-chronic pancreatitis with concomitant PE, similar to that reported in the studies by Fu et al. and Chung et al. [3,9], and, to the best of our knowledge, this is the first such case to be reported from Pakistan.CP is classified into chronic calcifying, chronic obstructive, and steroid-responsive pancreatitis [6]. In children, CP is caused by genetic mutations, autoimmune pancreatitis, gall stones, hyperlipidemia, congenital pancreatic abnormalities, trauma, toxins, inborn error of metabolism, as well as obstructive (biliary) and idiopathic causes [3]. In addition to caloric and macronutrient deficiencies, micronutrient deficiencies are also pertinent, particularly fat-soluble vitamins [7].

The management of CP predominately involves medical supportive therapy, with intravenous hydration, a fat-free diet, pain control, and pancreatic enzyme replacement. Gastric acid secretion is suppressed with proton pump inhibitors [3-7]. The treatment of autoimmune pancreatitis involves steroid therapy. The majority of the complications related to pancreatitis resolve spontaneously with medical supportive therapy, and surgical management is rarely required. In patients with intractable pain despite following an analgesic ladder and interventional procedures like ERCP sphincteroplasty and pancreaticojejunostomy (PJ), sub-total/total pancreatectomy and islet cell transplantation are performed in specialist centers as a last resort [8].

Mortality in CP mainly results from infection in children, and due to cardiovascular disease or malignancy in rare cases. Venous thromboembolism is a fatal complication of acute-on-chronic pancreatitis. Its pathogenesis is best described by Virchow’s triad, which includes increased blood coagulability, stasis, and endothelial damage. When DVT breaks, the embolus travels upwards, and, after passing through the heart, it can get stuck in narrow vessels of the lungs, resulting in PE. The diagnosis can be confirmed with the help of a D-dimer blood test, lung scan, CT angiography, echocardiography, MRI, and pulmonary angiography. Magnetic resonance cholangiopancreatography and endoscopic retrograding cholangiopancreatography are the techniques used to define the anatomy of the gland and are mandatory if surgery is being considered [9].

## Conclusions

Venous thromboembolism in patients with acute-on-chronic pancreatitis is a rare complication and is under-recognized; however, it can be fatal, and clinicians need to be extra vigilant when treating patients with pancreatitis. To avoid missed and incorrect diagnoses, clinicians should be mindful of the significance of dynamically monitoring blood coagulation indicators, such as D-dimer, during diagnosis and treatment. Preventive measures along with early screening can help clinicians improve patient outcomes and prevent mortality. This can be achieved by involving a multidisciplinary team and performing regular periodic assessments.
